# Application of Smart Insoles in Assessing Dynamic Stability in Patients with Chronic Ankle Instability: A Comparative Study

**DOI:** 10.3390/s25030646

**Published:** 2025-01-22

**Authors:** Seonghyun Kang, Jaewook Kim, Yekwang Kim, Juhui Moon, Hak Jun Kim, Seung-Jong Kim

**Affiliations:** 1 Department of Biomedical Engineering, Korea University College of Medicine, Seoul 02841, Republic of Korea; ss7186@korea.ac.kr (S.K.); jaewook_kim@korea.ac.kr (J.K.); yekwang@korea.ac.kr (Y.K.); answn520@korea.ac.kr (J.M.); 2 Department of Orthopaedic Surgery, Korea University Guro Hospital, Seoul 08308, Republic of Korea; dakjul@korea.ac.kr

**Keywords:** chronic ankle instability, smart insole, ankle functional tests, biomechanics, quantitative assessment

## Abstract

Chronic ankle instability (CAI), due to its chronic nature and biomechanical complexity, is well-suited for continuous monitoring and tele-rehabilitation using wearable sensor technology. This study assessed whether a smart insole system, equipped with 4 force-sensing resistor sensors and an inertial measurement unit, combined with functional tests and biomechanical indices, could distinguish CAI patients from healthy controls. A total of 21 CAI patients (23.8 ± 5.1 years) and 16 controls (22.62 ± 2.60 years) completed a battery of functional performance tests while wearing the smart insole system. The results showed an increased medial-lateral pressure ratio in the CAI during heel raise (*p* = 0.031, effect size = 0.82) and hop tests, suggesting an everted foot position. Significant deviations in center-of-pressure trajectory during double-leg heel raises (*p* = 0.005, effect size = 1.10) suggested asymmetric motion coordination, while compensatory fluctuations of the lifted limb during single-leg balance tests (*p* = 0.011, effect size = 1.03) were greater in CAI patients. These findings facilitated the development of features to characterize CAI-specific movement patterns. Together, this system shows promise as a quantitative assessment tool for CAI, supporting improved treatment outcomes through tele-rehabilitation.

## 1. Introduction

Lateral ankle sprains are the most common musculoskeletal injury in physically active populations [1,2]. Its incidence is likely higher than reported, as less than 50% of affected individuals seek medical attention [3,4]. While some individuals who experience ankle sprains may recover without any physical impairments, termed “copers”, it is common for relatively benign ankle sprains to lead to the development of chronic ankle instability (CAI), with prevalence rates ranging from 40 to 70% [5,6,7,8]. If not appropriately addressed, CAI can lead to osteoarthritis, osteochondral injury, and reduced joint range of motion [9].

Due to its chronic nature, CAI demands continuous functional assessment and long-term management to achieve optimal outcomes. Recent advancements in wearable sensor technology have created new opportunities, introducing innovative solutions such as tele-rehabilitation and continuous remote monitoring [10]. These technologies offer practical and accessible options for managing chronic musculoskeletal conditions, particularly for individuals with limited access to in-person care. By enabling the continuous tracking of disease severity and rehabilitation progress, facilitating self-assessment, and empowering healthcare providers to monitor progress remotely, these approaches enhance treatment outcomes and functional recovery. Furthermore, they redefine the paradigm of patient-centered care, making it more adaptive and accessible in the management of CAI.

In order to quantitatively assess the physical function of CAI patients, understanding their anatomical and biomechanical complexity is essential. While attenuation of the lateral talofibular ligaments is a primary cause of CAI, it is now recognized as a result of complex interactions involving patho-mechanical, sensory-perceptual, and motor-behavioral impairments [11]. This perspective extends beyond the traditional categories of mechanical and functional instability [12]. Comorbidities such as surrounding muscle weakness, joint capsule stiffness, osteophyte formation, and sensorimotor system deficits have often been observed [13,14,15,16]. Although these impairments are anatomically localized to the ankle, disruptions in functional biomechanics can have cascading effects, negatively impacting proximal joints, the contralateral limb, and whole-body balance [17,18]. These complexities underscore the necessity for a comprehensive approach to the rehabilitation and quantitative functional assessment of CAI.

Traditional, non-instrumented functional performance tests (FPTs), such as the Star Excursion Balance Test (SEBT), Dynamic Leap Balance Test (DLBT), hop tests, and weight-bearing lunge tests, are well-established tools for assessing physical performance [19,20]. These tests evaluate parameters like reach distance, performance time, angle, and error rates, offering a practical foundation for clinical assessments. Their advantages include being cost-effective, easy to administer, and widely accessible in both clinical and field settings [20]. Advancements in sensor technology, particularly with high-resolution systems such as motion capture and force plates, have further improved the analysis of movement dynamics, enabling researchers to quantify physical impairments with greater precision. Biomechanical features such as prolonged stabilization time during landing tasks [6,21,22], significant center-of-pressure (*COP*) fluctuations during balancing, and increased rearfoot inversion during gait [23] have been identified as key indicators for diagnosing and evaluating physical function.

Despite these technological advancements, both non-instrumented and instrumented FPTs remain unsuitable for tele-rehabilitation or remote monitoring due to inherent limitations. Non-instrumented tests often require professional supervision to ensure proper technique and posture, while the high cost and complexity of equipment of sensors make instrumented tests impractical for at-home use [24,25]. These challenges underscore the need for highly accessible solutions that bridge the gap between clinical assessments and remote monitoring capabilities. Wearable sensors, including inertial measurement units (IMUs) and plantar pressure sensors, as well as 2D or RGB-D cameras, provide a more practical alternative for remote monitoring and rehabilitation [26,27]. These technologies provide detailed biomechanical insights that often surpass those of non-instrumented assessments, while remaining cost-effective, portable, and user-friendly—qualities that make them particularly well-suited for tele-rehabilitation in CAI patients.

However, wearable sensors face certain limitations, such as reduced reliability and resolution compared to high-specification systems, which can limit their adoption in clinical settings. Addressing these challenges requires the careful identification of specific tasks that align with the strengths of these sensors while compensating for their weaknesses. Additionally, the development of standardized protocols and robust indices can enhance the reliability and practical application of these devices in CAI management. For instance, in our previous study [28], we demonstrated that the medial-lateral (ML) pressure ratio, measured using a smart insole system equipped with 4 force-sensing resistor (FSR) sensors, effectively detected ankle eversion during a heel-raise task. This system demonstrated comparable performance to a high-quality pressure sensor composed of 99 capacitive sensors in distinguishing ankle eversion. Interestingly, the simplicity of the sensor system not only facilitated its use but also enhanced its ability to make clearer distinctions.

**Figure 1:**
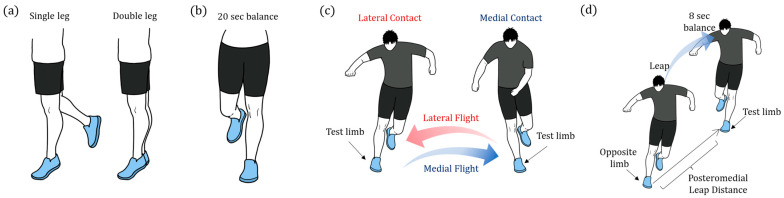
**Illustration of experimental procedures.** Four categories of selected functional assessment tasks are depicted: (**a**) Single-leg and double-leg heel raises. (**b**) Single-leg stance performed on the ground and foam, each lasting 20 s. (**c**) Side hop performed for each leg, involving four phases: lateral contact (LC), medial flight (MF), medial contact (MC), and lateral flight (LF). (**d**) Dynamic leap and balance involving jumping to the posteromedial direction with the opposite limb, landing with the test limb, balancing for 8 s, and leaping back to the initial position.

In this study, we selected specific tasks from conventional FPTs and rehabilitation exercises believed to be appropriate for the application of a smart insole system equipped with 4 FSR sensors and a 6-axis IMU sensor. We not only utilized features commonly examined in previous studies, such as *COP*, ML pressure, and frontal plane angles, but also introduced novel features that we have shown to characterize patients with CAI. It is posited that ipsilateral abnormalities can influence the contralateral limb, which further causes asymmetries in bilateral limb movements. Therefore, we propose a feature that can assess synchronicity during the double-leg heel raise (DHR). While the assessment of *COP* has traditionally been applied to measure single-leg balance, it may be possible to identify dynamic instability by observing the kinematics of the contralateral limb. This study aimed to ascertain the features, obtained from the smart insole system during a selected battery of functional tests (Figure 1), that could yield a novel approach for the quantitative functional assessment of patients with CAI.

## 2. Methods

### 2.1. Population

A total of 37 participants were enrolled in the study, including 21 CAI patients and 16 healthy controls. The participants recruited for our study were physically active college students who regularly participated in intramural basketball. A comprehensive diagnostic approach, including history-taking, physical examinations, and score assessments, was conducted equally for both feet. Subjects in the CAI group met all the following inclusion criteria: (1) history of a significant ankle sprain, (2) repetitive ankle sprains, (3) frequent reports of “giving way”, and (4) at least 2 scores above the recommended cutoff from three discriminative instruments. The three discriminative instrument questionnaires and their respective thresholds were applied according to a recent guideline [8]: an Identification of Functional Ankle Instability (IdFAI) score of 11 or greater, a Cumberland Ankle Instability Tool (CAIT) score of 25 or less, and a Foot and Ankle Ability Measure score of 85% or less. Participants were excluded if they had (1) bilateral instability, (2) acute/subacute symptoms of ankle sprain within the past 1 month, (3) a history of major operations or fractures in the lower extremities, or (4) were currently undergoing formal rehabilitation of the lower limb.

Before the sensor-based tests, physical examinations commonly used to identify baseline ankle functionality, including a talar tilt test, anterior drawer test, and isometric strength measurements of the ankle joint using a handheld dynamometer (ActivForce 2, ActivForce, San Diego, CA, USA), were conducted by either an orthopedic surgeon or a physical therapist. The baseline characteristics of the enrolled patients are shown in Table 1. All participants provided written informed consent, and research ethics for human experiments were ensured by conducting the sessions in accordance with the protocols approved by the Institutional Review Board of Korea University (IRB No.: 2022-0392-02, Approval date: 29 November 2022). All experiments were performed in accordance with approved guidelines and the principles of the Declaration of Helsinki.

### 2.2. Experimental Protocols

**Figure 2:**
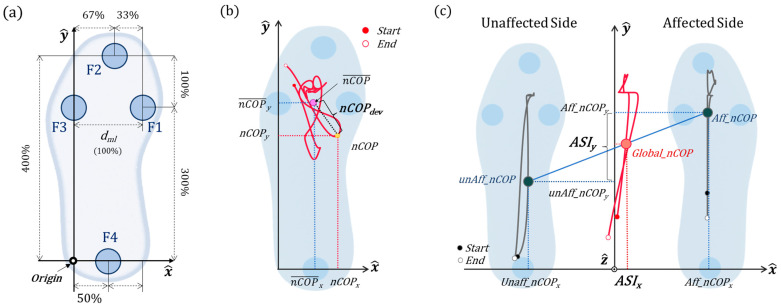
**Schematics of the smart insole and indices related to FSR signal.** (**a**) Layout of the four FSR sensors embedded in the smart insole system. The distance between F2 (toe) and F4 (heel) is four times the distance (*d_ml_*) between F1 (medial) and F3 (lateral) sensors. (**b**) The trajectory of nCOP is represented by a red line. $nCOP_{dev}$ is defined as the distance between the mean position of nCOP and nCOP of each data frame. (**c**) Definition and illustration of the asynchrony index (ASI). The global normalized *COP* (Global_nCOP), shown as a red line and circle, was calculated based on each limb’s nCOP (gray line and circle) and the ratio of foot pressure. $ASI_{x}$ is defined as the x-coordinate of Global_nCOP, and $ASI_{y}$ is determined by the difference between the *COP* y-coordinates of each limb ($nCOP_{y}$).

All the participants placed a pair of wireless smart insoles (NEUROGAIT Insole, Salted Ltd., Hanam-si, Republic of Korea) in place of their original insoles. Each smart insole provided real-time data acquisition from 4 FSR sensors and a 6-axis IMU sensor operating at 100 Hz. The sizes of the insoles were provided according to the user’s shoe size to ensure that the FSR sensors were embedded beneath the second toe (F2), heel (F4), and the first (F1) and fourth metatarsal heads (F3) (Figure 2). The six-axis IMU and processor were placed beneath the second and third metatarsal base, respectively.

This study aims to investigate whether data acquired from smart insoles can be processed into biomechanical features that characterize the movements of patients with CAI and potentially serve as discriminative markers to distinguish between patients with CAI and healthy controls. To facilitate the insoles’ clinical application in tele-rehabilitation, we prioritized tasks that can be performed independently without supervision, serving as both assessment and rehabilitation exercises. Thus, ankle functional assessment tasks appropriate for smart insole data collection were selected. These tests included single-leg heel raise (SHR), DHR, single-leg stance on the ground (SSG), single-leg stance on foam (SSF), dynamic leap and balance test (DLBT), and side-hop test (Figure 1).

Twelve repetitions of heel raise to maximal height were performed for both SHR and DHR, with each repetition held at maximal height for 1 s before coming down, while maintaining full knee throughout the task. During the SHR task, the subjects were permitted to lightly press against a bar (approximately chest height) with both index fingers to maintain balance. The lateral distance between the feet during the DHR task was set to be twice the width of the feet. For the SSG and SSF sessions, participants were instructed to place their hands on their waist and look forward for 20 s. Prior to performing the DLBT, we conducted a posteromedial directional SEBT for each limb and calculated the reach distance from the mean of three trials [29,30]. The landing target for the DLBT was placed at 120% of the mean SEBT reach distance to normalize the difficulty of the task for each participant [31]. After the leap phase, the participants were instructed to maintain single-leg balance for 8 s, which was repeated five times per limb. In tasks involving single-leg stance (SHR, SSG, SSF, and DLBT), the contralateral limb was flexed behind the stance leg. If the raised leg touched the ground, participants were instructed to immediately lift it again. This reflected the loss of balance, allowing for the interpretation of instability through increased fluctuations in the data. Side-hops were performed over a level ground surface marked with pieces of colored tape placed 30 cm apart. The participants were instructed to perform 10 roundtrip hops as quickly as possible. The sequence of each exercise was selected randomly, and a resting period lasting longer than 3 min was provided between each task. During tasks involving a unilateral stance limb, such as SHR, single-leg stances, and side hops, separate experiments were performed for each leg. All participants performed a few practice trials to familiarize themselves with the movements prior to data acquisition.

### 2.3. Data Processing and Analysis

The system provides 4 FSR signals, 3-axis acceleration, and 3-axis gyroscope signals per insole. According to the positions of the FSR sensors, the local forces at the medial, toe, lateral, and heel parts were determined using sensors F1 to F4, respectively. For the data acquired from the IMU sensor, the x-axis corresponds to ML, the y-axis to the anterior–posterior (AP), and the z-axis to the inferior–superior direction (Figure 2a). For the FSR and acceleration signals, previous comparisons with high-quality sensors revealed no significant drift or errors [28]. As a result, only a simple data smoothing technique, specifically a moving mean filter, was applied for signal correction. In contrast, sensor drift is a common issue for gyroscope signals, as errors can accumulate when integrating angular velocities to calculate orientation angles. To address this, we employed a polynomial fitting bias self-compensation method to ensure accurate orientation angle measurements [32].

The data from each task were segmented into individual trials. The start and end points of the heel raise events were identified by setting a simple threshold for y-axis acceleration (AccY). In this study, it was determined that the x- and y-directional acceleration signals increased proportionally with the roll and pitch angles, respectively, owing to the influence of gravitational acceleration when the IMU sensor was tilted slowly. For the analysis of the SSG and SSF, the initiation and termination of the tasks were determined by the lifting of the contralateral limb off the ground and placing it back down. These events were captured by the disappearance and reappearance of the FSR signal, respectively. For the DLBT, we only analyzed the post-landing and balancing phases of the involved limb where the signals from the FSR sensors appeared. The hop test was segmented using a straightforward threshold for the FSR signals of the involved limb, resulting in four distinct phases (Figure 1c). For all tasks, the segmented data were normalized with respect to time such that the data could be represented as a percentage of the task cycle. Subsequently, the ensemble means and standard deviations were calculated.

We calculated the medial partial pressure (*MPP*), which was the ratio of the medial FSR signal to the sum of the medial and lateral FSR signals. The *MPP* has already been proven to be a viable indicator of frontal posture and balance during the heel-raise task [28]. We also estimated the ML and AP COP coordinates using the FSR sensors. Although the actual insole sizes varied among the participants, the ratio between the heel-to-toe FSR sensor distance and the medial-to-lateral sensor distance (*d_ml_*) was maintained at 4:1. Therefore, we normalized the COP against *d_ml_* and defined it as the normalized *COP* (nCOP). The nCOP position for a single limb was calculated using the values from the 4 FSR sensors and their relative positions as shown in Equations (1) and (2). Furthermore, we measured the two-dimensional distances between the current nCOP and mean nCOP to track the nCOP during each functional task (Equation (3), Figure 2b).

(1) $$n{COP}_{x}\left(t\right)=\frac{F_{1}\left(t\right)+0.67\times F_{2}\left(t\right)+0.5\times F_{4}\left(t\right)}{\sum_{i=1}^{4}F_{i}\left(t\right)\;}\times 100$$

(2) $${nCOP}_{y}\left(t\right)=\frac{F_{2}\left(t\right)+0.74\times (F_{1}\left(t\right)+F_{3}\left(t\right))}{\sum_{i=1}^{4}F_{i}\left(t\right)\;}\times 400\;$$

(3) $${nCOP}_{dev}\left(t\right)=\sqrt{{\left(n{COP}_{x}\left(t\right)-\bar{n{COP}_{x}}\right)}^{2}+{\left(n{COP}_{y}\left(t\right)-\bar{{nCOP}_{y}}\right)}^{2}}\;$$

where $nCOP_{x}$ denotes the position of the nCOP in the ML direction (*x*-axis) and $nCOP_{y}$ represents the nCOP position in the AP direction (*y*-axis). $\bar{n{COP}_{x}}$ and $\bar{{nCOP}_{y}}$ denote the mean nCOP in the ML and AP direction, respectively. $F_{n}$ signifies the force detected by the nth FSR sensor, and t indicates the current sample. $nCOP_{dev}$ represents the deviation of nCOP from the mean nCOP.

In the DHR task, it is most desirable to perform the task symmetrically, such that the real-time position and magnitude of the *COP* are the same for both feet. However, in many cases, asymmetry and asynchrony are observed between affected and unaffected legs. To evaluate this discrepancy, we propose an asynchrony index (ASI) (Figure 2c). The *x*-direction component of ASI ($ASI_{x}$) is defined as the x-direction deviation of the global nCOP, as shown in Equation (4). A positive $ASI_{x}$ indicates that the center of mass was on the affected side, whereas a negative $ASI_{x}$ indicates the opposite. The *y*-direction component of ASI ($ASI_{y}$) was calculated from the difference between the nCOP of both feet, as shown in Equation (5). A positive $ASI_{y}$ indicates that the nCOP of the affected limb was closer to that of the toe than that of the unaffected limb. Finally, the *z*-direction component of ASI ($ASI_{z}$) was defined as the difference in the AccY signals of both feet, as shown in Equation (6). As previously described, during the heel raise, the AccY signal increases proportionally with the pitch angle of the foot. A positive $ASI_{z}$ indicates that the height of the raised heel on the affected side is greater.

(4) $${ASI}_{x}\left(t\right)=\frac{\sum_{i=1}^{4}{unAff_{F}}_{i}\left(t\right)\;\left(-200+un{Aff_{nCOP}}_{x}\left(t\right)\right)\;\;}{\sum_{i=1}^{4}{unAff_{F}}_{i}\left(t\right)+\sum_{i=1}^{4}{Aff_{F}}_{i}\left(t\right)\;}+\frac{\sum_{i=1}^{4}{Aff\_F}_{i}\left(t\right)\;\left(200-{Aff\_nCOP}_{x}\left(t\right)\right)\;\;}{\sum_{i=1}^{4}{unAff\_F}_{i}\left(t\right)+\sum_{i=1}^{4}{Aff\_F}_{i}\left(t\right)\;}$$

(5) $${ASI}_{y}\left(t\right)={Aff\_nCOP}_{y}\left(t\right)-un{Aff\_nCOP}_{y}\left(t\right)\;$$

(6) $${ASI}_{z}\left(t\right)=Aff\_accY\left(t\right)-unAff\_accY\left(t\right)$$

where ${Aff\_F}_{n}$ and ${unAff\_F}_{n}$ indicate the nth FSR signals from the unaffected and affected limbs within the CAI group, respectively. For the healthy group, these terms specifically denote the nondominant and dominant limbs, respectively. Aff_nCOP and unAff_nCOP represent the nCOP position vectors on the affected and unaffected sides, respectively, and Aff_accY and unAff_accY denote AccY measured by the IMU on each side.

**Figure 3:**
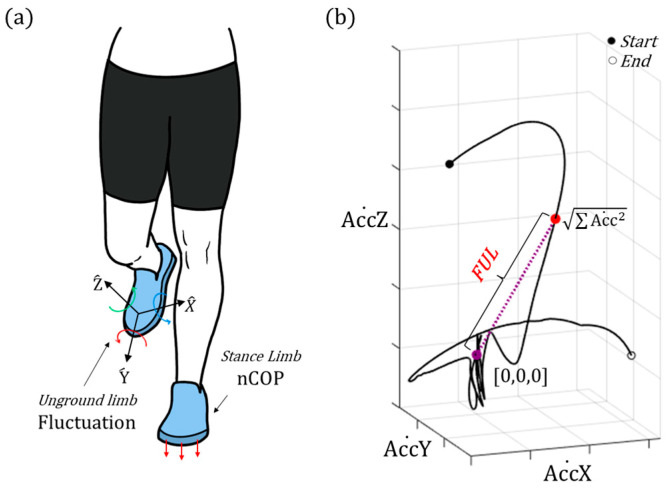
**Illustration of** FUL **and representative data.** (**a**) During the single-leg stance tasks, nCOP and FUL are monitored on the supporting foot and lifted foot, respectively. (**b**) An example of ${FUL}_{\dot{acc}}$ for one cycle (8 s) of a single-leg stance task.

To assess stability during single-leg balancing tasks, we propose new metrics that quantify the fluctuations of the ungrounded limb (FUL), as illustrated in Figure 3. FUL has two components calculated by differentiating the acceleration and angular velocity obtained from the IMU sensors (Equations (7) and (8)).

(7) $${FUL}_{\dot{acc}}\left(t\right)=\sqrt{{\dot{acc}X(t)}^{2}+{\dot{acc}Y(t)}^{2}+{\dot{acc}Z(t)}^{2}}$$

(8) $${FUL}_{gyro}\left(t\right)=\sqrt{{gyroX}^{2}+{gyroY}^{2}+{gyroZ}^{2}}$$

where $\dot{accX},\;\dot{\;accY},\;\;and\;\dot{accZ}$ represent the time derivatives of the *x*-, *y*-, and *z*-directional accelerations, respectively. *gyroX*, *gyroY*, and *gyroZ* are the angular velocities in the *x*-, *y*-, and *z*-directions, respectively.

The Symmetry Index (*SI*) [33] and ensemble means were used to represent phase-domain features. The normality of each dataset was assessed using the Shapiro–Wilk test. Based on the results, either independent *t*-tests or Wilcoxon rank-sum tests were employed to analyze differences between patients with CAI and the control group. The effect size for each variable was determined using the absolute value of Cohen’s d. All statistical analyses were conducted using SPSS version 27 (IBM Corp., Armonk, NY, USA).

## 3. Results

Table 1 displays the demographic information, clinical scores, ankle laxity test results, and isometric strength test values of the participants. No statistically significant differences were observed in age, body mass index, or sex distribution between the groups. Additionally, the isometric strength of the ankle joint, which was normalized by dividing each participant’s isometric strength by their weight, showed no significant variance between the affected limb of the patients with CAI and the dominant limb of the control group. However, all three validated questionnaires—CAIT, IdFAI, and FAMM-sport—depicted statistically significant differences, which implies that we have properly enrolled the CAI and healthy control participants.

### 3.1. Heel Raise Test

**Figure 4:**
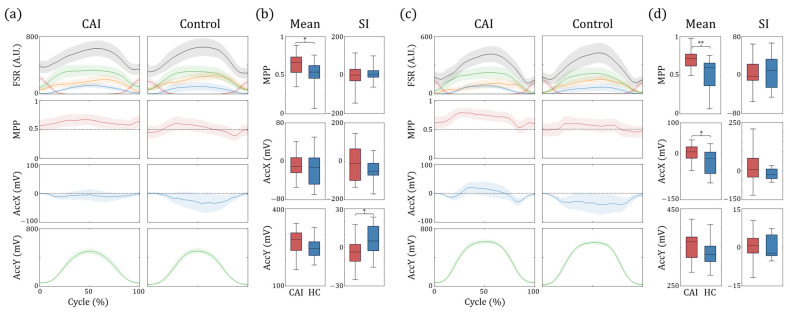
**Results of heel raise tasks.** Data from single-leg and double-leg heel raises are depicted in (**a**–**d**), respectively. In the first and second columns of (**a**,**c**), the lines represent the mean values, and shaded areas indicate standard errors. In the first row, the black line denotes the summation of values from the 4 FSR sensors, whereas the colored lines represent individual sensor outputs: green for F1 (medial), blue for F2 (toe), orange for F3 (lateral), and red for F4 (heel). Box plots in (**b**,**d**) illustrate the data distribution for the entire groups (* *p* < 0.05, ** *p* < 0.01). Data are presented as medians (black line in the box), 25th and 75th percentiles (boxes), and minimum and maximum values (whiskers). (HC: healthy control).

Figure 4a presents data from both the CAI and control groups during the SHR task, represented by lines and shaded areas that indicate mean values and standard errors, respectively. The x-direction acceleration (AccX) displayed in the third row represents the frontal plane motion of the foot, with negative and positive values corresponding to inversion and eversion, respectively. On the affected side of the patient group, an inclination towards the medial side can be observed, with an *MPP* value exceeding 0.5, accompanied by a flat AccX curve near 0. Conversely, in the dominant limbs of the control group, the *MPP* values fluctuated around 0.5, with a slightly inverted foot position. The quantitative group statistical analysis performed on the *MPP* values (CAI: 0.63 ± 0.15, Control: 0.49 ± 0.19, *p* = 0.031, effect size = 0.82) shows significant differences between patients and the control group (Figure 4b). While the SI values for *MPP* and AccX were statistically similar, we observed a significant difference in the AccY values (CAI: −3.44 ± 10.32, Control: 5.26 ± 11.55, *p* = 0.034, effect size = 0.79).

For the DHR task, *MPP* values increased more significantly in patients with CAI than in those with SHR (Figure 4c). Notably, when the pitch angle of the foot is small, the AccX data are negative, indicating foot inversion. However, when the heel rises above a certain height, it switches to foot eversion. This pattern was significantly different from that of the control group, in which AccX remained negative throughout the cycle. Statistical analysis revealed significant increases in mean *MPP* (CAI: 0.70 ± 0.13, Control: 0.53 ± 0.20, *p* = 0.008, effect size = 1.01) and AccX (CAI: −0.68 ± 26.20, Control: −28.67 ± 40.05, *p* = 0.032, effect size = 0.83), while no differences were observed in the SI (Figure 4d).

**Figure 5:**
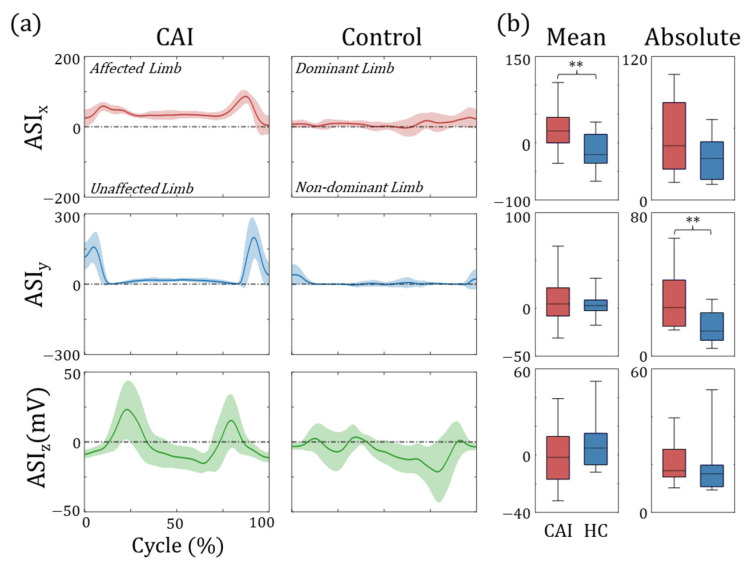
**Results of** ASI**.** (**a**) Representative plots containing mean (line) and standard deviation (shaded area) for one subject from the patient and control groups. (**b**) Box plots showing the distribution of the ASI values for all participants, including the independent *t*-test or Wilcoxon rank-sum test results (** *p* < 0.01).

Figure 5 presents representative plots of the ASI values during the DHR task for subjects in the CAI and control groups. In the patient data, a positive $ASI_{x}$ was observed throughout the entire phase, indicating that weight was consistently shifted towards the affected side. For $ASI_{y}$, there were two positive peaks, where the first peak indicated that, at the beginning of the heel raise, the *COP* of the affected leg moved towards the toes ahead of that of the unaffected leg. The second peak suggests that the heel of the unaffected leg hit the ground prior to that of the affected side. In contrast, the $ASI_{y}$ of the healthy control group remained near 0 throughout the phase, indicating that both feet were well synchronized. Statistical analysis identified significant differences in the mean $ASI_{x}$ (CAI: 24.08 ± 38.39, Control: −14.17 ± 30.73, *p* = 0.005, effect size = 1.10), indicating that weight was generally shifted to the affected side for patients with CAI (Figure 5b). Moreover, the absolute values of $ASI_{y}$ (CAI: 32.22 ± 16.32, Control: 16.64 ± 8.77, *p* = 0.002, effect size = 1.19) were significantly higher in the patient group, while mean $ASI_{y}$ values did not show any differences. This suggests that, although there was no consistent pattern of balance shifting to one side, the degree of AP directional imbalance was notably larger in the patient group.

### 3.2. Single-Leg Stance Test and DLBT

**Figure 6:**
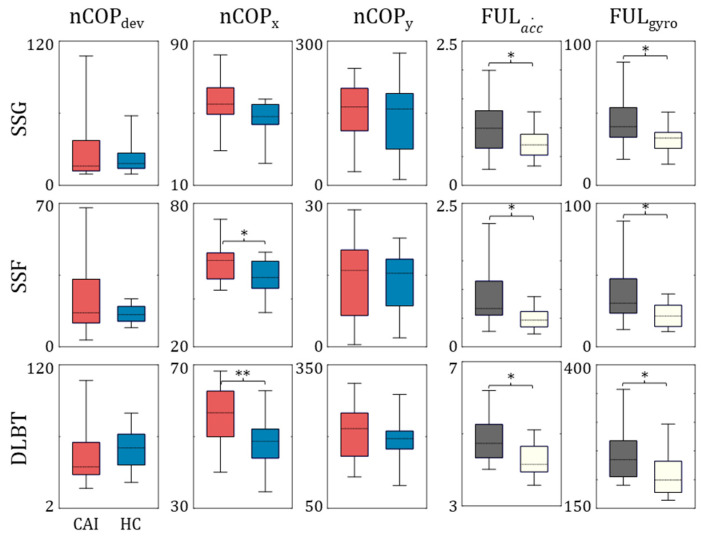
**Results of single-leg stance and DLBT tasks.** The results of group analysis, including data distribution and statistical evaluations for tasks associated with single-leg stance (SSG, SSF, and DLBT), are presented as box plots (* *p* < 0.05, ** *p* < 0.01).

The SSG, SSF, and DLBT tasks were analyzed using the nCOP trajectory and FUL indices, which were calculated from the FSR signals of the supporting foot and IMU signals of the lifted foot, respectively, as shown in Figure 6. Larger values for both nCOP and FUL indicated increased postural fluctuations, suggesting decreased stability and whole-body balance control. The level of difficulty for SSF and DLBT is expected to increase due to the soft foam and dynamic leap, respectively. For nCOP, we only observed significant differences in $nCOP_{x}$ values for both SSF (CAI: 55.18 ± 7.83, Control: 48.43 ± 8.15, *p* = 0.044, effect size = 0.84) and DLBT (CAI: 54.02 ± 8.59, Control: 45.27 ± 8.33, *p* = 0.011, effect size = 1.03), while significant increases were observed for $FUL_{\dot{acc}}$ across all three tasks, with relatively larger values for DLBT (CAI: 4.82 ± 0.63, Control: 4.27 ± 0.49, *p* = 0.021, effect size = 0.97) compared to SSG (CAI: 0.86 ± 0.47, Control: 0.54 ± 0.21, *p* = 0.030, effect size = 0.88) and SSF (CAI: 1.01 ± 0.44, Control: 0.72 ± 0.28, *p* = 0.049, effect size = 0.79). $FUL_{gyro}$ values for SSG (CAI: 45.69 ± 18.98, Control: 32.12 ± 10.65, *p* = 0.023, effect size = 0.88), SSF (CAI: 37.31 ± 19.35, Control: 22.14 ± 8.76, *p* = 0.011, effect size = 1.01), and DLBT (CAI: 246.81 ± 50.38, Control: 208.23 ± 39.21, *p* = 0.023, effect size = 0.85) also showed statistical differences.

### 3.3. Side Hop Test

**Figure 7:**
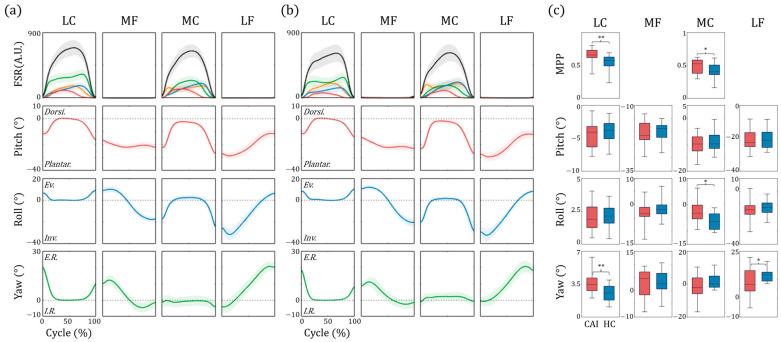
**Results of side-hop task.** (**a**,**b**) display representative plots for sequential phases of the side-hop task for the patient and control group, respectively. The lines represent mean values, and shaded areas indicate standard errors. In the first row, the black line represents the summation of values from the 4 FSR sensors, whereas the colored lines denote individual sensor values: green for F1 (medial), blue for F2 (toe), orange for F3 (lateral), and red for F4 (heel). The second, third, and fourth rows represent the calculated angle data from the gyroscope sensors. Box plots illustrating the results of statistical analyses (* *p* < 0.05, ** *p* < 0.01) are shown in (**c**). (Dorsi.: dorsi-flexion, Plantar.: plantarflexion, Ev.: eversion, Inv.: inversion, E.R.: external rotation, I.R.: internal rotation).

Figure 7a,b illustrates the 4 phases during the side hop in both the patient and control groups. The CAI group exhibited less weight-shifting in the ML direction than the healthy controls during the lateral contact (LC) phase. During the medial contact (MC) phase, we observed a more rapid shift from lateral to the medial foot pressure immediately after landing for patients with CAI. In addition, patients with CAI showed a later shift back to the lateral side prior to the lift off part of the MC phase. Statistical analysis (Figure 7c) showed significant increases in *MPP* for the patient group during both the LC (CAI: 0.643 ± 0.125, Control: 0.535 ± 0.133, *p* = 0.007, effect size = 0.84) and MC (CAI: 0.486 ± 0.110, Control: 0.398 ± 0.104, *p* = 0.023, effect size = 0.82). The *SI* values also highlighted significant differences between LC (CAI: 30.09 ± 47.53, Control: −10.73 ± 36.27, *p* = 0.017, effect size = 0.97) and MC (CAI: 5.75 ± 35.05, Control: −18.94 ± 18.03, *p* = 0.020, effect size = 0.89).

We primarily analyzed the angular data displayed in the second, third, and fourth rows of Figure 7. The roll angles during the post-landing (0–30%) and pre-hopping (70–100%) phases of the MC were slightly lower in the control group, suggesting a relatively more inverted posture. We also observed an increase in the yaw angle from the late medial flight phase to the early lateral flight (LF) phase in the control group. This suggests a more internally rotated foot during this period in contrast to the increased external rotation observed in the late LF and early LC phases. These differences were also evident in the statistical analysis: roll angle for the MC phase (CAI: −2.96 ± 4.58, Control: −6.29 ± 3.30, *p* = 0.037, effect size = 0.83) and yaw angle in the LC (CAI: 3.57 ± 1.07, Control: 2.54 ± 0.39, *p* = 0.006, effect size = 1.28) and LF phases (CAI: 8.26 ± 6.93, Control: 12.32 ± 3.69, *p* = 0.042, effect size = 0.73). Although some subjects in the patient group showed reduced pitch and roll angles during the landing phase of hopping, indicating inadequate preparation for landing, or exhibited an opposite pattern of angular data during the flight phases, these differences were not statistically significant.

## 4. Discussion

In this study, we investigated the potential application of an affordable smart insole system to quantitatively assess ankle functionality and identify patients with CAI. This approach could be applied for remote monitoring in clinical settings. Six test configurations consisting of modified traditional FPTs and rehabilitation tasks were administered to 21 young CAI patients and 16 healthy controls. Participants wore smart insoles that measured foot pressure distribution, as well as 3-axis acceleration and 3-axis angular velocity from both feet. The results demonstrate that features extracted from the smart insoles can effectively detect and quantify the altered biomechanical characteristics associated with CAI. Furthermore, the indices newly defined in this study, such as ASI for DHR tasks and FUL for single-leg stance tasks, were found to be useful for functional assessment.

### 4.1. Heel Raise Test

The heel raise task requires adequate muscle strength, endurance, and proprioceptive skills to maintain balance [34]. These characteristics make the heel raise a suitable method for assessing ankle function and a valuable rehabilitation tool for foot–ankle complex disorders, especially for CAI. Therefore, identifying biomechanical properties and quantitatively monitoring the heel raise motion can provide significant benefits for assisting in diagnosis and improving treatment outcomes. As a first step towards the clinical application of this concept, we conducted a study to demonstrate the potential of a smart insole system for detecting correct postures during heel raise exercises [28]. In this study, we examined distinct biomechanical features of patients with CAI.

During the SHR and DHR tasks, we observed increased *MPP* and AccX in the CAI group, indicating that the weight was concentrated in the medial aspect of the foot with a slightly everted posture. This result diverges from that of a previous study that reported increased rearfoot inversion during SHR tasks among delivery workers with CAI [35]. The authors suggest that their results align with the increased inversion often observed during the toe-off phase in patients with CAI due to similar biomechanics. However, previous studies have reported medially concentrated pressure during the heel-off phase, suggesting that greater rearfoot eversion reduces lateral ligament strain [36,37]. Moreover, we believe that the gait and heel raise movements differ significantly because, during the lift phase of the heel raise, the extremities must bear the entire body weight, whereas the ground reaction force decreases during plantar flexion during gait. This distinction was further elicited in the heel raise tasks because we instructed the participants to hold the maximum height position. The progression of ankle inversion, indicated by a decrease in AccX, was observed in the initial heel-raising phase (0–20%) for both groups (Figure 4c). While inversion was maintained in the control group, a transition to eversion occurred during the later rising and holding phase (30–70%), followed by inversion during the fall phase for the CAI group. An inverted hindfoot results in a locked subtalar joint, which is biomechanically advantageous for maintaining stability and enabling effective delivery of plantar flexion forces from the calf muscle during the lifted heel posture [38,39]. However, an increased inversion moment to the ankle joint can be challenging for patients with CAI who exhibit decreased neuromuscular control of the inverted posture and attenuated function of the peroneus longus [40], which resists the inversion moment during heel raises, whether isometrically or eccentrically. Moreover, repeating the heel raise task 12 times may exacerbate this condition; however, further analysis is required to substantiate these hypotheses.

Alterations in a single joint or segment can significantly influence the biomechanics of the adjacent structures, thereby affecting the entire kinematic chain, including the contralateral limb. Owing to the chronic nature of CAI, extended compensatory adaptations might result in adhesion to an unbalanced posture and functional deficits such as diminished hip strength and tightened muscles [1,41,42]. This exacerbates the asymmetry of bilateral limb kinematics. We posit that this asymmetry is more pronounced when both feet are required to perform the same movement simultaneously. Quantitative assessment of this asymmetry could aid in the diagnosis and monitoring of general physical function in individuals with CAI. To our knowledge, no previous studies have explored the distribution of weight across bilateral limbs when conducting symmetrical motions such as two-legged jumps, stands, or DHR in patients with CAI. An increase in the $ASI_{x}$ in the CAI group suggests a predominant weight shift toward the affected limb. A shift in the global center of mass toward the affected side reduces the angle between the center of mass and the ankle joint, thereby diminishing the frontal plane moment and the ML directional force exerted on the joints. Furthermore, an imbalance in hip joint abductor strength could contribute to this phenomenon as a hip domain balancing strategy becomes prevalent in individuals with CAI [22]. A leaning posture leads to relative adduction of the hip joint, shortening the moment arm of the hip abductors, a modification that may enhance the functionality of these weakened muscles in patients with CAI to maintain ML balance. The absolute value of $ASI_{y}$ in the CAI group also increased significantly, suggesting asynchronous heel lift progression. Both the $ASI_{x}$ and $ASI_{y}$ values demonstrate high effect sizes of 1.10 and 1.19, respectively, indicating their effectiveness as indices for reflecting the biomechanical characteristics of CAI.

### 4.2. Single-Leg Stance Test and DLBT

The measurement of the trajectory and degree of sway of the *COP* is commonly employed to assess balance or physical function using foot pressure sensors or force plates. The overall compensatory actions for maintaining global balance, which involve the trunk, upper extremities, and contralateral limbs, are consequently aggregated and expressed as fluctuations in the *COP*. In this study, we aimed to capture and quantify compensatory motions for balancing the strategies of the contralateral limb using an IMU sensor embedded in a smart insole. This method could provide additional biomechanical insights for detecting deficits in balance function. Moreover, it may prove to be a more robust tool under certain conditions than *COP* measurements, particularly in scenarios where the contact surface between the foot and ground is narrow or uneven, leading to insufficient sensor data.

The experimental results indicated a significant increase in both the $FUL_{\dot{acc}}$ and $FUL_{gyro}$ in the CAI group across all three tasks, whereas *COP* fluctuation was only apparent in the ML direction ($nCOP_{x}$) of the SSF and DLBT, as shown in Figure 4d. These findings highlight the efficacy of FUL in capturing the balancing function. Additionally, the higher FUL values observed in the more dynamic movement of the DLBT compared to the SSF or SSG indirectly suggest that these FUL values effectively capture the body sway. The high effect sizes of FUL values, ranging from 0.79 to 1.01, further highlight its utility in assessing physical function, particularly in individuals with CAI.

However, there are limitations, and there is a need for further biomechanical studies. The acceleration values obtained from the IMU sensors contained gravitational acceleration; thus, we could not truly assess the acceleration of the foot. Thus, the derivative of the acceleration utilized for calculating $FUL_{\dot{acc}}$ does not indicate jerk. To establish a more physiologically meaningful interpretation, such as quantifying variances in pure linear acceleration and excluding the influence of gravity, further investigation should be conducted. Moreover, further studies involving other sensors, such as cameras, and different types of patient groups with balance deficits are planned.

### 4.3. Side-Hop Test

Strong evidence supports the use of single-limb hopping under timed conditions to evaluate physical impairments in CAI. A systematic review and meta-analysis revealed high effect sizes for timed-hop (mean effect size = −1.056, *p* = 0.009), side-hop (mean effect size = −2.314, *p* = 0.001), and multiple-hop tests (mean effect size = 1.399, *p* < 0.001), underscoring their performance in assessing functional deficits [20]. Hop movements serve as both an assessment tool and a rehabilitation exercise, making their quantitative analysis through wearable sensors a promising approach for tele-rehabilitation. Among the various hop tests, we selected the side-hop test to focus on frontal plane function. While alterations in landing and side-cutting mechanisms among CAI patients are well documented, biomechanical analyses using the side-hop test remain limited [43,44,45]. To the best of our knowledge, this study is the first to employ insole-type sensors for such an analysis, paving the way for new approaches in functional assessment.

We observed an abrupt weight shift towards the medial side in the MC phase and a continued medial-predominant status in the LC phase in patients with CAI, causing a significantly increased *MPP*. This medial-dominant weight distribution likely decreased the inversion torque load on the exterior ankle joint, which we interpret as a compensatory strategy to reduce the burden on the lateral talofibular ligament complex and surrounding structures. This postulation corresponds well with previous investigations [43], which reported reduced inversion moments of the ankle joint during side-hop tests using force plates with motion capture systems.

Consistent with the *MPP* results, we also observed an increase in the roll angle, which indicated decreased inversion during the MC phase for patients with CAI. However, because the gyroscope sensor is only installed inside the insole, the estimated angles represent the relative orientation of the foot in the global frame and not the relative tibia-foot angle. Thus, the displayed patterns and absolute values of foot orientation may differ from those of previous kinematic studies [44,45] that reported the actual ankle joint angles. Although no studies have reported the kinematics along the axial plane of motion, we observed significant differences in the yaw angle. In patients with CAI, increased external rotation in the post-landing state during the LC phase was noted, providing advantages for activating the peroneus longus muscle. The eccentric contraction of this muscle can resist excessive inversion during lateral cutting.

Among the meaningful features identified in the side-hop test, the yaw angle during the LC phase showed the highest effect size at 1.28. While traditional timed-hop tests, which assess physical function based on repetition number, demonstrated higher effect sizes [20], our feature also produced comparable results. Performing timed-hop tests independently can pose challenges, as capturing failure trials or determining whether the foot lands beyond a specific distance can significantly affect test results. Utilizing wearable sensors not only simplifies the measurement of repetitions but also enables detailed analysis of movement variations during the task. This added capability positions wearable sensors as a promising tool for enhancing the assessment and understanding of functional performance.

### 4.4. Limitiations

This study has several limitations. Firstly, the sample size and composition were limited. Based on G-Power 3.1.9.7 calculations (significance level 0.05, statistical power 95%, two-tailed), 23–35 participants per group are required to detect an effect size of 0.8–1.0 for independent *t*-tests or Wilcoxon rank-sum tests. Our study enrolled fewer participants than the calculated numbers, which warrants caution when interpreting the statistical results. Additionally, biomechanical characteristics vary across age groups, and this study included only active young participants. To achieve broader clinical significance, future studies should involve a larger number of participants across a wider demographic range.

Secondly, although this study identified significant differences in the extracted features between CAI and control groups, it did not establish whether these features can effectively reflect disease progression or severity. To enable practical applications, it is essential to validate our indices’ ability to quantitatively represent disease severity or functional impairment through direct comparisons with well-established, documented tests in the field. As an initial step, this study highlighted indices that clearly differentiate between groups and offered insights into their biomechanical characteristics. However, further research is required to conduct detailed analyses and evaluate whether this system can be effectively utilized for tele-rehabilitation in clinical settings.

## 5. Conclusions

Our findings indicate that individuals with CAI exhibit distinct biomechanical characteristics that can be distinguished by performing appropriate physical assessments using smart insole systems. Notably, novel methods that utilize perturbations from the lifted limb and 3-axis symmetry indices during DHR tasks have highlighted significant differences between the patient and control groups. Beyond understanding these altered mechanisms, it is crucial to consider their accessibility and usability in real-world clinical and rehabilitation settings. The smart insole system used in this study is wireless, making it suitable for field deployment without substantial modification. Consequently, we believe our research could serve as a foundation for advancing tele-rehabilitation, assisting physicians in monitoring home-based exercises, and furthermore development of software as a medical device via biofeedback mechanisms.

## Figures and Tables

**Table 1:** Subject Characteristics.

	CAI (*n* = 21)	Control (*n* = 16)	*p* Value ^b^
**Demographics**			
Sex (male/female)	15/6	11/5	0.860
Age	23.76 ± 5.09	22.62 ± 2.60	1.000
BMI	22.29 ± 2.57	22.34 ± 3.31	0.818
**Clinical Scores**			
CAIT ^a^	20.52 ± 4.45	27.56 ± 2.19	<0.001
IdFAI	19.24 ± 5.58	6.94 ± 6.18	<0.001
FAMM-sport (%)	88.33 ± 13.43	97.62 ± 4.99	0.004
**Ankle Laxity Test (+/−)**			
Anterior drawer test	2/21	0/16	
Posterior drawer test	0/21	0/16	
Talar tilt test	3/21	0/16	
**Strength (N/Kg)**			
Inversion	1.89 ± 0.53	1.91 ± 0.46	0.855
Eversion	1.69 ± 0.44	1.79 ± 0.35	0.652
Plantar flexion	2.65 ± 0.51	2.52 ± 0.38	0.211
Dorsi flexion	2.57 ± 0.43	2.53 ± 0.41	0.907

^a^ Affected side of the CAI group and dominant side of the control group. ^b^ Statistical analyses were conducted using chi-square tests for categorical variables and either independent *t*-tests or Wilcoxon rank-sum tests for continuous variables.

## Data Availability

The data that supports the findings of this study are available upon request to the corresponding author.
